# Story of a surgery nurse

**DOI:** 10.3325/cmj.2011.52.580

**Published:** 2011-08

**Authors:** Binezija Kolesar

**Affiliations:** Surgery Ward, Vukovar Medical Center, Vukovar, Croatia

I was born in Belgrade in 1939, in a Croatian-Bosniak Muslim family. I lived in Vukovar since 1941. After graduating from the College for Nursing in Osijek, I worked in Vukovar Medical Center since 1961. In 1962, I was appointed the head nurse of the Surgery Ward.

I grew up in Vukovar, made my family and career there and there is no other town for me but Vukovar. Now, at the age when I should enjoy in what I have accomplished and earned, I am here in an empty hotel room in a strange city, without any material possessions. I came here in my summer dress and sandals. I will never get over the fact that we had to leave Vukovar, but I know I will go back.

## The beginning

For me and my fellow citizens, the war started on May 2, 1991, with the massacre of Croatian guardsmen in Borovo Selo. Until August 25, the wounded were brought to the hospital every day, indicating that fights were going on close to Vukovar. We never expected such destruction and terror but realized that we had to prepare for a total war. The Crisis Management Council of the hospital was established in July. We decided to make supplies of food, drugs, dressing material - everything we might need to survive. We received donations from all over the world. In the beginning, we thought they were unnecessary – we could not believe that the war could be so brutal and that we would soon run out of everything. Shelters were also prepared; Dr Matoš organized the cleaning and sanitation of never used anti-atom bomb (AA) shelter. By August 25, when an extensive air attack was launched on Vukovar, the shelter was hardly usable. It was located near the new building of the hospital. The patients from the old part (Psychiatry, Ophthalmology, Neurology and Pediatrics Wards) went down to the basement. After August 25, air-raids were not anymore announced and the general danger warning was in effect the whole time, and the hospital started living and working in the shelters. While the communication with Vinkovci was still open, the gravely wounded were transported there. This was the only way to create place for the wounded we expected, as the shelters were relatively small.

## The last two months

For the last two months we were under complete siege and the inflow of the wounded increased rapidly. I remember days when more than 80 newly wounded were admitted. The working conditions became worse and worse. The hospital was under continuous attacks and it was impossible to leave its premises.

One of my duties was to find beds for the wounded. It was not easy at all, since there was a shortage of not only beds, but also blankets, sheets, pajamas, slippers and other necessary accessories. We still managed while some of us were able to bring things from their homes.

The lightly injured were transferred to Borovo, where our medical unit was organized in the *Borovokomerc* building. We knew it was not the best solution and that communications might easily be cut off, but we had no choice at the moment. For the last two weeks, no information came back to us from there and I do not know what happened there.

We lived in the underground darkness and the only sounds from the outside were those of crash and rumble from destroyed houses and falling trees. Improvisation was the only way of resisting the aggressor. Yugoslav Federal Army (YFA) and Serbian paramilitary forces wanted to occupy Vukovar at any cost, even total destruction. We knew that, but we never lost our spirit.

There was enough food in the beginning. The National Guard soldiers helped us. Everybody helped and brought food from their homes. Toward the end, we could not get anything because people could not go out of the shelters. In the morning, we had tea and a piece of toast, or only tea; lunch consisted of beans, stew, paste or potatoes, and dinner of rice or grits.

Water shortage was the worst. Access to the water wells was dangerous. A half a liter of water was our daily allowance. Some even managed to wash hair with that. But most of the time we were thirsty.

Water from the central heating system was used. Electricity was out and only one generator worked. It was used mainly for x-ray machine. It was cold.

The shelter was directly hit by grenades several times. Dr Bosanac's husband, engineer by profession, helped us with the repairs. Most of the hospital building was destroyed, with only remains of the construction left.

## The evacuation

Despite the courage and good organization of the defense, the National Guard ran out of ammunition. We knew the end was near.

On Wednesday, November 20, we were supposed to be evacuated in the presence of the European Community Mission and the whole world, but the YFA and Serbian paramilitary did not let that happen. They took 200 wounded, all men who did not belong to the medical staff. Husbands of nurses, who were in the shelter, were arrested. My husband was also taken away in that chaos. Everybody tried to save his family from the bloodthirsty paramilitaries. It was a sad sight to watch our former colleagues, neighbors and classmates walking around with lists and searching for men to take away. Many never returned. We do not know what happened to them. Our journey from the hospital was a horrible ordeal – buses halted every 100 m, waited for several hours, then returned. We were manipulated and humiliated. My mother died on the way.

